# Abscisic Acid Treatment in Patients with Prediabetes

**DOI:** 10.3390/nu12102931

**Published:** 2020-09-24

**Authors:** Giuseppe Derosa, Pamela Maffioli, Angela D’Angelo, Paola S. Preti, Giancarlo Tenore, Ettore Novellino

**Affiliations:** 1 Department of Internal Medicine and Therapeutics, IRCCS Policlinico San Matteo Foundation, University of Pavia, 27100 Pavia, Italy; pamelamaffioli@hotmail.it (P.M.); pretipa05@unipv.it (P.S.P.); 2 Laboratory of Molecular Medicine, University of Pavia, 27100 Pavia, Italy; labmedmol@smatteo.pv.it; 3 Department of Pharmacy, University of Naples Federico II, 80131 Napoli, Italy; giancarlo.tenore@unina.it (G.T.); ettore.novellino@unina.it (E.N.)

**Keywords:** abscisic acid, prediabetes, dysglycemia, treatment, plasma glucose

## Abstract

Aim: to evaluate the effects of abscisic acid (ABA), contained in dwarf peaches, on the regression of impaired fasting glucose (IFG) or impaired glucose tolerance (IGT) conditions. Materials and methods: sixty-five patients with IFG or IGT were randomized to take ABA or placebo for 3 months. We evaluated: fasting plasma glucose (FPG), postprandial plasma glucose (PPG), glycated hemoglobin (HbA_1c_), fasting plasma insulin (FPI), homeostatic model assessment of insulin resistance (HOMA-IR), lipid profile and high sensitivity C-reactive protein (Hs-CRP). At baseline, and after 3 months, all patients underwent an oral glucose tolerance test (OGTT), an euglycemic hyperinsulinemic clamp, and a glucagon test. Results: a significant reduction of HbA_1c_, FPG, PPG, FPI and HOMA-IR was observed in the ABA group. After 3 months, 26.7% of patients returned to a normal glycemic status in the ABA group versus zero patients in placebo group; 20.0% were classified as IFG and 53.3% as IGT in the nutraceutical group versus 33.3% and 63.3% in the placebo group. The M value was higher in the ABA group at the end of the treatment. Finally, Hs-CRP was reduced after 3 months of ABA consumption. Conclusions: abscisic acid can be effective in ameliorating glyco-metabolic compensation and in reducing inflammatory status in patients with IFG or IGT.

## 1. Introduction

Prediabetes is a disease state preceding the onset of diabetes and comprises impaired fasting glucose (IFG) or impaired glucose tolerance (IGT) conditions. The main pathophysiology features of IFG and IGT are insulin resistance and a reduced functionality of the pancreatic β-cells [1].

According to American Diabetes Association guidelines, dysglycemia is defined by a fasting plasma glucose ≥ 100 and <126 mg/dL or by a glycated hemoglobin (HbA_1c_) ≥ 5.7% but <6.5% [2]. In these cases, we should perform an oral glucose tolerance test (OGTT) with 75 g of glucose and evaluate glycemia at 0 and 120 min from the load to define patient glycemic status: if glycemia after 120 min is ≥200 mg/dL, type 2 diabetes mellitus (T2DM) will be diagnosed, if glycemia after 120 min is ≥140 mg/dL but <200 mg/dL, IGT will be diagnosed, if glycemia after 120 min is <140 mg/dL, IFG will be diagnosed [3].

It has been observed that almost all type 2 diabetic patients go across the prediabetes phase, whose average duration is about 10 years [4]. During this long transition state, it is possible to avoid the evolution towards diabetes if proper interventions are adopted. In subjects with prediabetes, the treatment goal should be the return to an euglycemic condition. At this regard, it has been showed that restoration and maintenance of normal glycemia values during prediabetes and early stages of T2DM can determine a long-term remission [5].

Several trials showed that one of most promising approaches to achieve this goal is a comprehensive lifestyle change consisting of diet, physical activity and weight loss [6], that, ameliorating β-cell function and insulin sensitivity, can contribute to prevent the onset of diabetes [7]. Although lifestyle intervention is often difficult to implement and maintain in the long-term because of poor adherence by patients [8,9], it represents the primary tool for prevention of T2DM development from prediabetes due to its efficacy, safety and cost-effectiveness [6].

It has also been reported that some nutraceuticals are effective and safe in improving insulin sensitivity and glycemic control in patients with dysglycemia [10,11,12].

Recently, abscisic acid (ABA) has aroused a considerable interest due to its involvement in the management of glucose homeostasis in humans [13]. Abscisic acid is a phytohormone that in addition to being present in various fruits and vegetables is produced and released from human pancreatic β-cells in response to high glucose concentrations. Moreover, at nanomolar concentrations, ABA regulates insulin secretion, intensifying its glucose-dependent release and stimulating a glucose-independent one [14].

One study showed that hyperglycemia produced an increase of ABA plasma levels in healthy subjects subjected to an OGTT and that this phytormone, at nanomolar concentrations, promoted peripheral glucose uptake in adipocytes and myoblasts, similarly to insulin [15].

It has also been observed that an alterated increase of ABA plasma concentration, after OGTT, occurs in T2DM and in gestational diabetes. However, in the latter, the glucose-induced rise of ABA levels has normalized after childbirth because of restoring of normal glucose tolerance [16].

Moreover, in healthy subjects, the consumption of ABA, present in fig fruit extracts, determined a significant decrease in postprandial glucose and insulin levels [17].

Overall, these results indicate that ABA, due to its glycemic-lowering activity, may play a pivotal role in physiology and in disorders of glucose homeostasis in humans.

The aim of this study was to evaluate the effects of ABA, contained in dwarf peaches, on the regression of IFG or IGT conditions and on glyco-lipid metabolism in IFG or IGT patients.

## 2. Materials and Methods

### 2.1. Study Design

This 3-month, double-blind, randomized, placebo-controlled, clinical trial was conducted at the Department of Internal Medicine and Therapeutics, University of Pavia and Department of Pharmacy, University of Naples Federico II, Italy. The study protocol was approved by the institutional Ethical Committee (P-2017000837) and was conducted in accordance with the 1994 Declaration of Helsinki [18], and its amendments and the Code of Good Clinical Practice. All patients provided written informed consent to participate in this study after a full explanation of the study.

### 2.2. Patients

We enrolled patients with IFG or IGT, not taking hypoglycemic agents (both pharmaceutical or nutraceutical agents). Suitable patients, identified from review of case notes and/or computerized clinic registers, were contacted by the investigators in person or by telephone.

Patients were excluded if they had type 1 diabetes mellitus or T2DM, impaired hepatic function (defined as plasma aminotransferase and/or gamma-glutamil transpeptidase (γ-GT) level higher than the three times the upper limit of normal (ULN) for age and sex), impaired renal function (defined as serum creatinine level higher than the ULN for age and sex), or gastrointestinal disorders; current or previous evidence of ischemic heart disease, heart failure, or stroke; weight change of >3 kg during the preceding 3 months; malignancy, and significant neurological or psychiatric disturbances, including alcohol or drug abuse. Excluded medications (within the previous 3 months) included hypoglycemic agents, laxatives, β-agonists (other than inhalers), cyproheptadine, antidepressants, antiserotoninergics, phenothiazines, barbiturates, oral corticosteroids, and antipsychotics. Women who were pregnant or breastfeeding or of childbearing potential and not taking adequate contraceptive precautions were also excluded.

### 2.3. Treatments

Patients were randomized to placebo or ABA (2 g, lyophilized) for 3 months. Both ABA and placebo were self-administered three times a day, 1 sachet before the breakfast, 1 sachet before the lunch, and 2 sachets before the dinner.

Both ABA and placebo were supplied as identical sachets with codes to ensure the blind status of the study. Randomization was done using a drawing of envelopes containing randomization codes prepared by a statistician. Medication compliance was assessed by counting the number of empty sachets returned at the time of specified clinic visits. Throughout the study, we instructed patients to take their first dose of new medication on the day after they were given the study medication. At the same time, all unused medication was retrieved for inventory. All medications were provided free of charge.

### 2.4. Assessments

At the study start, all patients underwent an initial screening assessment including a medical history, physical examination, vital signs (blood pressure and heart rate), a 12-lead electrocardiogram, measurements of height and body weight, calculation of body mass index (BMI), evaluation of fasting plasma glucose (FPG), postprandial plasma glucose (PPG), HbA_1c_, fasting plasma insulin (FPI), homeostatic model assessment of insulin resistance (HOMA-IR), total cholesterol (TC), low density lipoprotein-cholesterol (LDL-C), high density lipoprotein-cholesterol (HDL-C), triglycerides (Tg), aspartate aminotransferase (AST), alanine aminotransferase (ALT), gamma-glutamyl transpeptidase (γ-GT), creatinine, and high sensitivity C-reactive protein (Hs-CRP).

All parameters were evaluated at baseline and after 3 months since the study start. Moreover, at baseline, and after 3 months, patients underwent an oral glucose tolerance test (OGTT), an euglycemic hyperinsulinemic clamp, and a glucagon test. For a description of how various parameters were assessed, please see our previous studies [19,20].

### 2.5. Oral Glucose Tolerance Test

Oral glucose tolerance tests were performed before randomization, and at the end of the study. Normal physical activity was allowed over the previous 3 days. No smoking was allowed during the test. The patients were placed in a quiet room and did not move from their location for the duration of the test. All patients drank a water glass of 200 mL in which 75 g of glucose had been diluted over a period of 5 min in the morning, between 08.00 and 09.00 h after a 12-h fast, and after dietary assessment to ensure a carbohydrate intake > 150 g/day over the previous 3 days [2]. Blood glucose samples were collected in EDTA-containing tubes (Becton Dickinson, Meylan Cedex, France) through a venous catheter from an antecubital vein immediately before and at 30, 60, 90, 120, 150, and 180 min after the glucose load. On the basis of the results recorded at 120 min after the OGTT, we diagnosed patients as euglycemic or as being affected by IFG, IGT or T2DM.

### 2.6. Euglycemic Hyperinsulinemic Clamp Technique

Euglycemic hyperinsulinemic clamps [21] were performed before randomization, and at the end of the study. Between 09.00 and 09.30 h, after the patients had fasted for 12 h overnight, an indwelling cannula (18-gauge polyethylene cannula; Venflon, Viggo, Helsingborg, SWEDEN) was placed into an antecubital vein for infusion of glucose and insulin. To obtain arterialized venous blood samples, an indwelling catheter was inserted in a retrograde fashion into a dorsal hand or wrist vein and maintained in a heated box at 70 °C. In the contralateral arm, a second cannula was introduced anterogradely in an antecubital vein of the forearm for the variable infusion of 20% glucose (L.I.M., Biondustria, Novi Ligure, AL, Italy) and insulin (1 mU min^−1^ kg^−1^, Humulin R, Eli Lilly, Indianapolis, IN, USA, using a Terumo microinfusion pump, TE-371 TIVA, Terumo Corporation, Tokyo, Japan). Arterialized blood samples were collected every 5 min to determine glucose concentration (EML 105, Radiometer, Copenhagen, Denmark). The amount of glucose infused was adjusted to maintain euglycemia at 90 mg/dL. During the euglycemic hyperinsulinemic clamp, the M-value was calculated based on the last 30 min (steady state) and after adjustments for steady-state insulin concentration (M/I).

### 2.7. Glucagon Stimulation Test Technique

Glucagon test were performed before randomization, and at the end of the study. Between 08.00 and 08.30 h, Glucagon (Novo Nordisk A/S, Bagsværd, Denmark) was injected into the antecubital vein (1 mg) within 2 min after an overnight fast. Patients did not receive any medication in the morning of the study. Blood samples were taken from the other arm at baseline (time 0) and after 6 min from the glucagon injection. Glycemia and C-peptide were evaluated at baseline and after the test. C-peptide is a biologically inactive peptide formed when β-cells convert proinsulin to insulin. The amount of C-peptide in the blood is directly related to the amount of insulin secreted by β-cells: abnormally low amounts of C-peptide in the blood suggest that insulin production is too low (or absent), and abnormally high amount can suggest insulin-resistance [22].

### 2.8. Statistical Analysis

We conducted an intention-to-treat (ITT) analysis in patients who had received ≥1 dose of study medication and had a subsequent efficacy observation. Patients were included in the tolerability analysis if they had received ≥1 dose of trial medication after randomization and had undergone a subsequent tolerability observation. We used a two-way repeated measures analysis of variance (ANOVA) to test continuous variables. Intervention effects were adjusted for additional potential confounders using analysis of covariance. We conducted an analysis of variance to assess the significance within and between groups. The null hypothesis that the expected mean glycemia change from the end of the study did not differ significantly between placebo, and nutraceutical was tested using a two-way repeated measures analysis of variance (ANOVA) model. Similar analyses were applied to the other variables. A 1-sample t-test was used to compare values obtained before and after treatment administration; 2-sample *t*-tests were used for between-group comparisons [23]. Statistical analysis of data was performed using the Statistical Package for Social Sciences software version 14.0 (SPSS Inc., Chicago, IL, USA). Data are presented as mean (SD). For all statistical analyses, *p* < 0.05 was considered statistically significant.

## 3. Results

### 3.1. Study Sample

**Figure 1:**
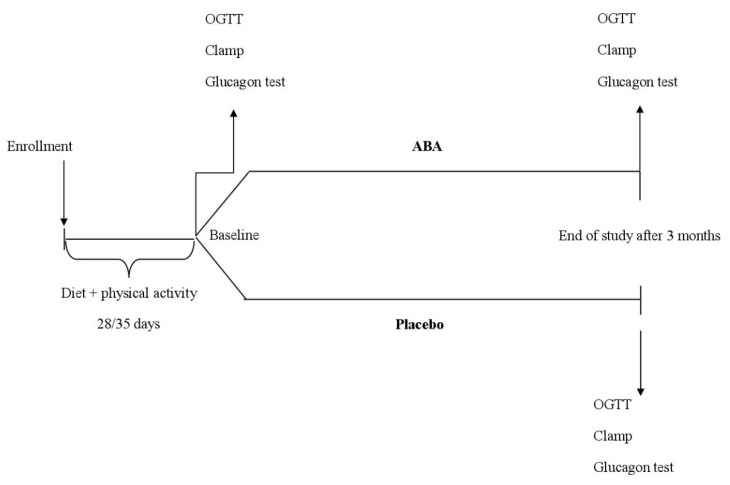
Study design. OGTT: oral glucose tolerance test; Clamp: euglycemic hyperinsulinemic clamp; ABA: abscisic acid.

A total of 65 patients were enrolled in the trial. Of these, 33 were randomized to ABA, and 32 to placebo. Sixty patients completed the study; there were five patients who did not complete the study and the reasons for premature withdrawal included noncompliance to treatment (one male in the ABA group, and one female in the placebo group, respectively) or lost to follow-up (one male in the placebo group and two females in the ABA group, respectively) (Figure 1). The characteristics of the patient population at the time of the start and during the study are shown in Table 1 and Table 2.

### 3.2. Anthropometric Parameters

No change was observed in BMI and circumferences in both treatments (Table 2).

### 3.3. Glyco-Metabolic Parameters

A significant decrease in FPG, PPG, FPI, and HOMA-IR was observed in the ABA group compared to the baseline value (*p* < 0.05 vs. baseline) and compared to the placebo group (*p* < 0.05 vs. placebo). The HbA_1c_ value was significantly reduced compared to baseline (*p* < 0.05 vs. baseline) in the group being treated with ABA (Table 2).

### 3.4. Lipid Profile

No significant modification was observed in the lipid profile parameters, although a slight nonsignificant reduction was seen in TC, LDL-C and Tg in the ABA group (Table 2).

### 3.5. Inflammation Parameter

Hs-CRP decreased significantly from baseline (*p* < 0.05 vs. baseline) in the ABA group and also compared to the placebo group (*p* < 0.05 vs. placebo) (Table 2).

### 3.6. OGTT Results

At baseline, 39.4% of patients were affected by IFG in the ABA group vs. 34.4% in placebo (*p* not significant), while 60.6% of patients were affected by IGT in the ABA group, and 65.6% in placebo group (*p* not significant). After 3 months, 26.7% of patients returned to a normal glycemic status in the ABA group vs. zero patients in placebo group (*p* < 0.05); at the end of the study, 20.0% were classified as IFG in the ABA group vs. 33.3% in placebo group (*p* < 0.05). In the ABA group, 53.3% were classified as IGT vs. 63.3% in the placebo group (*p* < 0.01). In the placebo group, 6.7% developed T2DM vs. zero patients in the ABA group (Table 1).

### 3.7. M Value during Euglycemic Hyperinsulinemic Clamp

The M value obtained after ABA treatment was higher with respect to baseline (*p* < 0.001 vs. baseline). No significant variations were recorded in the placebo group with respect to baseline. Furthermore, M values recorded with ABA were higher than the one observed with placebo (*p* < 0.0001).

Considering normal insulin sensitivity as an M value ≥ 7.5 mg/kg/min, at the end of the study, more patients returned to normal insulin sensitivity (89%) with the ABA treatment with respect to placebo. Moreover, 11% of patients reached an M value ≥ 4 and <7.5 mg/kg/min, and zero patients had an M value < 4 mg/kg/min (value of insulin resistance) at the end of the study in the ABA group, respectively (Table 3).

### 3.8. Glucagon Test Results

As expected, there was a significant increase in FPG and C-peptide after 6 min from the glucagon injection, at the baseline in ABA group, and in the placebo group (*p* < 0.01 for FPG and *p* < 0.001 for C-peptide vs. time 0, respectively). At 3 months, the glucagon test was repeated and there was a significant increase in FPG at 6 min (*p* < 0.01 vs. time 0), reduced compared to baseline (*p* < 0.05 vs. baseline), but significant compared to the placebo group (*p* < 0.05 vs. placebo) in the ABA group, while FPG and C-peptide were significantly increased at 6 min with an increase similar to that obtained with the same baseline glucagon test (*p* < 0.01 for FPG and *p* < 0.001 for C-peptide vs. time 0, respectively) in the placebo group (Table 4 and Table 5).

### 3.9. Safety and Treatment Acceptance

No significant changes in transaminases, γ-GT or creatinine were recorded during the study. Considering a score among one and ten, where one is the worst, and ten is the best, no differences were recorded between groups regarding acceptance of treatment that was well tolerated.

## 4. Discussion

The present study showed that ABA is effective in improving glyco-metabolic and inflammation parameters in patients with IFG or IGT.

Recently, Magnone et al. demonstrated that chronic consumption of a supplement containing a low dose of ABA ameliorated the prediabetes markers (FPG −30.2% and HbA_1c_ −8.1%) in subjects with borderline values of FPG (≥100 mg/dl) and HbA_1c_ (≥5.7%) [24] defined in the American Diabetes Association (ADA) recommendations for prediabetes [2]. It has been also proven that the improvement was greater in these subjects than in healthy ones, suggesting the beneficial effect of low-dose ABA supplementation in prediabetics. The authors obtained similar results in mice nourished with a high glucose content diet and treated with synthetic ABA at a concentration equal to that present in the supplement, reporting a significant HbA_1c_ reduction (around −63.0%) [24]. In a previous study, Magnone et al. showed that ABA at a low dose ameliorated the glycemic profile and reduced insulin levels in rats and healthy adults. The absence of increased insulin secretion was probably due to the stimulation by ABA of muscle glucose uptake and the greater sensitivity of human GLUT4-expressing cells to ABA than pancreatic β-cells [25].

Moreover, it has been reported that in healthy subjects, the consumption of two different doses of ABA added to glucose drink produced, for the higher one, a significant decrease of PPG and insulin levels (approximately −25%) while, for the lower dose, a significant reduction only in postprandial insulin concentration (about −15%) with a dose-response lowering for both ABA amounts. The lower dose decreased, but not significantly, PPG levels by around 14% [17].

Our data are consistent with the above results regarding a decrease in FPG of 4.9 mg/dL (−4.5%), in PPG of 13.9 mg/dL (−9.7%), in HbA_1c_ of 0.4% (−6.8%), in FPI of 1.1 μU/mL (−10.7%) and in HOMA-IR of 0.41 (−14.6%) in prediabetic patients who took ABA.

Guri et al. demonstrated, in obese and prediabetic mice, that the involvement of ABA in regulating glucose metabolism is due to its structural similarity to thiazolidinediones and its efficacy similar to that of these antidiabetic oral drugs [26]. Nevertheless, unlike thiazolidinediones, ABA exerts its hypoglycemic action, in mammals, by binding lanthionine synthetase C-like 2 (LANCL2) and acting on peroxisome proliferator-activated receptor gamma (PPARγ) with a mechanism independent of the receptor ligand-binding domain [27,28].

Magnone et al. showed that in subjects, in which ABA ameliorated prediabetes markers, its consumption also improved metabolic syndrome parameters (BMI −4.5%, WC −9.0%, and TC −19.3%) [24]. In fact, these subjects had borderline values of BMI (≥25 kg/m^2^), WC (≥88 cm in female and ≥102 cm in males) and TC (>200 mg/dL) established by ATP III guidelines for metabolic syndrome [29]. As seen for prediabetes markers, the percentage reduction of these parameters was higher in these subjects with respect to those obtained in healthy ones. Furthermore, in high-glucose fed mice, ABA intake results in a significant lowering of body weight (−25.0%), TC (approximately −7.0%) and Tg (about −21.0%), as reported in humans [24].

In our prediabetic patients, the ABA treatment did not determine any change in anthropometric parameters as well as in lipid profile with the exception of a slight, but not significant, reduction in TC (−4.1 mg/dL, −1.9%), LDL-C (−3.5 mg/dL, −2.4%) and Tg (−4.8 mg/dL, −3.9%) which, however, is indicative of a downward trend.

In this study, ABA supplementation has been shown significantly to reduce Hs-CRP levels (−0.3 mg/L, −23.1%), thus improving the inflammatory status in overweight prediabetic patients. Diabetes and obesity share low-degree chronic systemic inflammation and insulin resistance. ABA intake has been demonstrated to reduce inflammation in obese and prediabetic mice. In this murine model, ABA consumption at the lowest dose determined a decrease of FPG and insulin levels with an improvement of glucose tolerance. The histological analysis of white adipose tissue also showed a decrease of adipocyte hypertrophy and macrophage infiltration in adipose tissue in addition to a down-regulation of tumor necrosis factor-α (TNF-α) mRNA expression [26,30].

Considering the euglycemic hyperinsulinemic clamp, our current data confirm what we have demonstrated in previous studies involving different nutraceuticals [10,11], testifying that it is possible to improve insulin resistance. In fact, 36.7% of patients reported normal blood sugar (<100 mg/dL) and an improvement in measured insulin sensitivity at the end of the study, as is known, with the M value. None of patients treated with the nutraceutical had a worsening of insulin resistance.

After the administration of glucagon, ABA gave a lower increase in FPG and a greater increase in C-peptide after 6 min compared to the values recorded during the glucagon stimulation test performed at the beginning of the study in the placebo group. Regarding the physiological mechanism through which this can happen, some reports showed that ABA stimulates glucose uptake in the muscle and increases the sensitivity of human GLUT4-expressing cells and stimulates human pancreatic β-cells [25]. This result is in line with our previous assessment of another insulin-sensitizing phytotherapy compound such as Berberis aristata [31].

Of course, our study has some limitations. We need to observe these changes in a longer period of observation and to verify the possible reversible effect after the interruption of the treatment. A final consideration is the limited, though statistically correct, number of patients in the study, hoping for trials with a higher number of patients.

## 5. Conclusions

Abscisic acid can be effective in ameliorating glyco-metabolic compensation and in reducing inflammatory status in patients with IFG or IGT.

## Figures and Tables

**Table 1:** Baseline, and 3-month general data of patients during abscisic acid (ABA) treatment and placebo.

Parameters	ABA		Placebo	
	Baseline	3 Months	Baseline	3 Months
Patients (*n*)	33	30	32	30
M/F	15/18	14/16	16/16	15/15
Age (years)	51.9 ± 6.5	-	52.2 ± 6.8	-
Smoking status (M/F)	7/6	6/5	8/6	6/6
IFG (*n*; %)	6/7 (39.4)	3/3 (26.7)	5/6 (34.4)	4/5 (30.0)
IGT (*n*; %)	9/11 (60.6)	3/10 (46.7)	11/10 (65.6)	10/9 (63.3)
EU from IFG (*n*; %)	-	5/3 (61.5)	-	0/0
EU from IGT (*n*; %)	-	0	-	0/0
IFG from IGT (*n*; %)	-	0/0	-	1/0 (4.8)
IGT from IFG (*n;* %)	-	3/0 (23.1)	-	0/0
D from IFG (*n*; %)	-	0/0	-	0/0
D from IGT (*n*; %)	-	0/0	-	1/1 (9.5)
Lost to FU from IFG (*n*; %)	-	1/1 (15.4)	-	0/1 (9.1)
Lost to FU from IGT (*n*; %)	-	0/1 (7.7)	-	1/0 (4.7)

Data are expressed as number (*n*) or percentage (%). M: males; F: females; IFG: impaired fasting glycemia; IGT: impaired glucose tolerance; EU: euglycemia; D: diabetes; FU: follow-up.

**Table 2:** Baseline, and 3-month anthropometric and biochemical parameters of patients during abscisic acid (ABA) treatment and placebo.

Parameters	ABA		Placebo	
	Baseline	3 Months	Baseline	3 Months
Height (cm)	1.69 ± 0.05	-	1.68 ± 0.04	-
Weight (kg)	77.4 ± 6.1	76.5 ± 5.9	77.1 ± 5.9	76.2 ± 5.7
BMI (kg/m^2^)	27.1 ± 1.3	26.8 ± 1.1	27.3 ± 1.5	27.0 ± 1.2
WC (cm)	86.5 ± 4.9	86.4 ± 4.8	86.8 ± 5.0	86.7 ± 4.9
HC (cm)	89.2 ± 5.2	89.0 ± 5.0	88.9 ± 4.9	88.7 ± 4.7
AC (cm)	97.2 ± 5.8	97.0 ± 5.6	97.4 ± 6.0	97.2 ± 5.8
FPG (mg/dL)	109.4 ± 6.5	104.5 ± 6.1 *^,^^	112.8 ± 5.6	110.7 ± 5.5
PPG (mg/dL)	144.0 ± 12.8	130.1 ± 12.8 *^,^^	153.3 ± 18.4	149.7 ± 18.5
HbA_1c_ (%)	5.9 ± 0.4	5.5 ± 0.2 *	5.8 ± 0.3	5.7 ± 0.2
FPI (μU/mL)	10.3 ± 6.7	9.2 ± 5.8 *^,^^	10.1 ± 6.5	10.5 ± 6.9
HOMA-IR	2.80 ± 0.7	2.39 ± 0.4 *^,^^	2.84 ± 0.8	2.89 ± 0.9
TC (mg/dL)	215.1 ± 15.8	211.0 ± 14.2	218.6 ± 16.9	220.2 ± 18.1
LDL-C (mg/dL)	146.9 ± 18.4	143.4 ± 17.7	150.9 ± 19.2	152.6 ± 20.7
HDL-C (mg/dL)	43.8 ± 5.0	44.0 ± 5.1	43.6 ± 4.9	43.7 ± 4.8
Tg (mg/dL)	122.1 ± 24.2	117.3 ± 22.0	120.4 ± 23.5	119.5 ± 23.1
AST (UI/L)	18.8 ± 10.8	18.5 ± 10.4	18.2 ± 10.3	18.4 ± 10.5
ALT (UI/L)	28.3 ± 14.2	28.9 ± 14.5	26.8 ± 13.1	26.1 ± 12.8
γ-GT (UI/L)	24.5 ± 8.1	24.1 ± 7.7	25.8 ± 8.7	25.3 ± 8.4
Creatinine (mg/dL)	0.6 ± 0.2	0.7 ± 0.3	0.7 ± 0.3	0.8 ± 0.4
Hs-CRP (mg/L)	1.3 ± 0.5	1.0 ± 0.2 *^,^^	1.3 ± 0.5	1.4 ± 0.6

Data are expressed as mean ± standard deviation. * *p* < 0.05 vs. baseline; ^ *p* < 0.05 vs. placebo. M: males; F: females; BMI: body mass index; WC: waist circumference; HC: hip circumference; AC: abdominal circumference; FPG: fasting plasma glucose; PPG: postprandial plasma glucose; HbA_1c_: glycated hemoglobin; FPI: fasting plasma insulin; HOMA-IR: homeostatic model assessment of insulin resistance; TC: total cholesterol; LDL-C: low density lipoprotein-cholesterol; HDL-C: high density lipoprotein-cholesterol; Tg: triglycerides; AST: aspartate aminotransferase; ALT: alanine aminotransferase; γ-GT: gamma-glutamyl transpeptidase; Hs-CRP: high sensitivity C-reactive protein.

**Table 3:** M value variation during the study.

	N	Baseline	End of Treatment	Delta End of Treatment vs. Baseline
ABA	33	6.09 ± 0.51	7.38 ± 0.89 *^,^°	1.29 ± 0.59 *
Placebo	32	6.02 ± 0.37	6.03 ± 0.76	0.01 ± 0.006

Data are expressed as mean ± standard deviation. * *p* < 0.001 vs. baseline; ° *p* < 0.0001 vs. placebo. ABA: abscisic acid. Definition of insulin sensitivity: Normal insulin sensitivity: M value ≥ 7.5 mg/kg/min; Impaired glucose tolerance: M value ≥ 4 and <7.5 mg/kg/min; Insulin resistance: M value < 4 mg/kg/min.

**Table 4:** Glucagon test at the baseline, and after 3 months in the abscisic acid (ABA) group.

	Baseline		3 Months	
	Time 0	6 min	Time 0	6 min
FPG (mg/dL)	110.6 ± 7.3	148.6 ± 21.3 ^	103.8 ± 5.9 *^,£^	131.2 ± 16.1 ^^,^*^,£^
C-peptide (ng/mL)	7.15 ± 2.38	20.45 ± 7.59 °	9.02 ± 4.72 *^,£^	31.07 ± 10.15 °^,$,§^

Data are expressed as mean ± standard deviation. ^ *p* < 0.01 vs. time 0; ° *p* < 0.001 vs. time 0; * *p* < 0.05 vs. baseline; ^$^ *p* < 0.01 vs. baseline; ^£^ *p* < 0.05 vs. placebo; ^§^ *p* < 0.01 vs. placebo. FPG: fasting plasma glucose.

**Table 5:** Glucagon test at the baseline, and after 3 months in the placebo group.

	Baseline		3 Months	
	Time 0	6 min	Time 0	6 min
FPG (mg/dL)	111.5 ± 7.9	146.1 ± 20.2 ^	112.1 ± 8.4	148.5 ± 21.4 ^
C-peptide (ng/mL)	7.28 ± 2.51	21.33 ± 7.91 °	7.02 ± 2.37	20.15 ± 6.88 °

Data are expressed as mean ± standard deviation. ^ *p* < 0.01 vs. time 0; ° *p* < 0.001 vs. time 0. FPG: fasting plasma glucose.
